# Supplementary material for: Dissolution Kinetics of International Simple Glass and Formation of Secondary Phases at Very High Surface Area to Solution Ratio in Young Cement Water
Source: Materials (Basel). 2021 Mar 6;14(5):1254. doi: 10.3390/ma14051254 (PMC7961375; doi:10.3390/ma14051254)
Supplement: Supplementary file 1 [file materials-14-01254-s001.pdf]

materials

MDPI

SUPPLEMENTARY

Dissolution Kinetics of International Simple Glass and Formation of Secondary Phases at very High Surface Area to Solution Ratio in Young Cement Water

Karine Ferrand [1,*], Martina Klinkenberg [2], Sébastien Caes [1], Jenna Poonoosamy [2], Wouter Van Renterghem [3], Juri Barthel [4], Karel Lemmens [1], Dirk Bosbach [2] and Felix Brandt [2]

[1] Institute for Environment, Health and Safety, SCK CEN, B-2400 Mol, Belgium; sebastien.caes@sckcen.be (S.C.); karel.lemmens@sckcen.be (K.L.)
[2] Institute of Energy and Climate Research (IEK-6): Nuclear Waste Management and Reactor Safety, Forschungszentrum Jülich GmbH, 52425 Jülich, Germany; m.klinkenberg@fz-juelich.de (M.K.); j.poonoosamy@fz-juelich.de (J.P.); d.bosbach@fz-juelich.de (D.B.); f.brandt@fz-juelich.de (F.B.)
[3] Institute for Microstructural and Non-destructive Analysis, SCK CEN, B-2400 Mol, Belgium; wouter.van.renterghem@sckcen.be
[4] Ernst Ruska-Centre (ER-C 2), Forschungszentrum Jülich GmbH, 52425 Jülich, Germany; ju.barthel@fz-juelich.de
* Correspondence: karine.ferrand@sckcen.be

Citation: Ferrand, K.; Klinkenberg, M.; Caes, S.; Poonoosamy, J.; Van Renterghem, W.; Lemmens, K.; Bosbach, D.; Brandt, F. Dissolution Kinetics of International Simple Glass and Formation of Secondary Phases at very High Surface Area to Solution Ratio in Young Cement Water. *Materials* **2021**, *14*, 1254. https://doi.org/10.3390/ma14051254

Academic Editor: Jian-Zhong Jiang

Received: 14 February 2021
Accepted: 2 March 2021
Published: 6 March 2021

Publisher's Note: MDPI stays neutral with regard to jurisdictional claims in published maps and institutional affiliations.

Copyright: © 2021 by the authors. Licensee MDPI, Basel, Switzerland. This article is an open access article distributed under the terms and conditions of the Creative Commons Attribution (CC BY) license (http://creativecommons.org/licenses/by/4.0/).

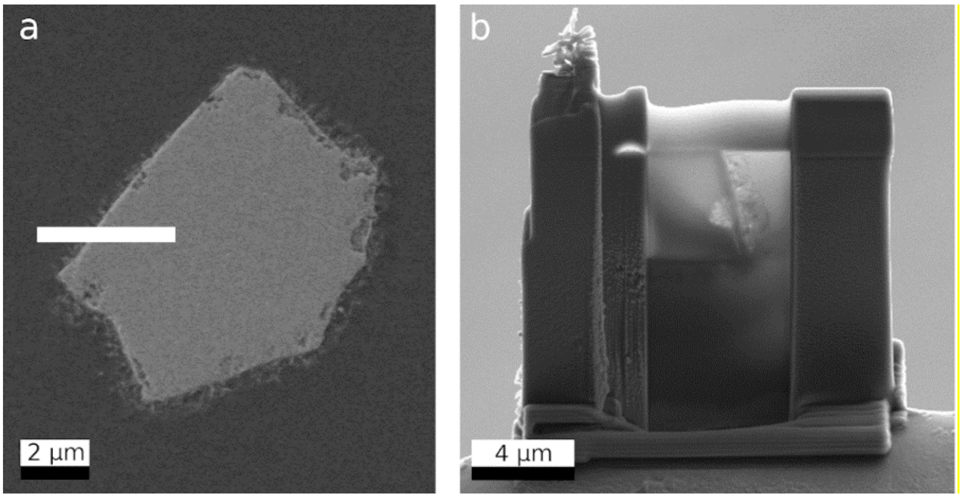

Figure S1. (**a**) SEM secondary electron image of the altered glass grain chosen for the FIB-TEM investigation; (**b**) SEM secondary electron image of the lamella in cross-section view. The dimension of the thinned area was 5.6 × 12.6 µm. The bright part on top of the lamella corresponds to the protective carbon layer.

Table 1. Temporal evolution of the pH and chemical composition of the aqueous solution. Concentrations were corrected for evaporation and for the calculation of NL_i the composition of the YCWCa was also taken into account. The uncertainties at 2 sigma on the elemental concentrations and NL_i values are ~10% and ~25%, respectively.

Data	Unit	Time (Days)				
		**59**	**288**	**385**	**632**	**952**
$pH_{(25\ °C)}$	-	11.20	11.14	10.97	10.79	10.78
$pH_{(70\ °C)}$	-	10.00	9.94	9.77	9.59	9.58
B	mg/L	5201.0	7868.9	6969.4	5674.6	5971.6
$B_{corr.evaporation}$	mg/L	5055.4	5712.8	6077.3	5674.6	5935.8
NL_B	g/m^2	0.36	0.40	0.43	0.40	0.42
Na	mg/L	9355.8	14,020.9	12,807.1	10,359.8	10,713.8
$Na_{corr.evaporation}$	mg/L	9093.9	10,179.2	11,167.8	10,359.8	10,713.8
$Na_{corr.YCWCa}$	mg/L	5973.9	7059.2	8047.8	7239.8	7593.8
NL_{Na}	g/m^2	0.25	0.30	0.34	0.30	0.32
Si	mg/L	1743.3	884.2	723.7	541.3	629.4
$Si_{corr.evaporation}$	mg/L	1694.5	641.9	631.1	541.3	629.4
NL_{Si}	g/m^2	0.02	0.01	0.01	0.01	0.01
Al	mg/L	0.12	0.11	0.12	0.17	2.93
$Al_{corr.evaporation}$	mg/L	0.11	0.08	0.10	0.17	2.93
$Al_{corr.YCMCa}$	mg/L	0.05	0.02	0.04	0.11	2.87
NL_{Al}	g/m^2	6.21×10^{-6}	2.71×10^{-6}	5.15×10^{-6}	1.34×10^{-5}	3.36×10^{-4}
Ca	mg/L	2.61	7.15	7.83	7.48	7.32
$Ca_{corr.evaporation}$	mg/L	2.54	5.19	6.83	7.48	7.32
$Ca_{corr.YCWCa}$	mg/L	negative values				
NL_{Ca}	g/m^2	-	-	-	-	-
K	mg/L	5407.9	4155.0	4048.1	3514.1	2693.1
$K_{corr.evaporation}$	mg/L	5256.5	3016.6	3529.9	3514.1	2693.1
Zr	µg/L	8.91	2.91	0.59	1.76	-
$Zr_{corr.evaporation}$	µg/L	8.66	2.1	0.51	1.76	-
NL_{Zr}	g/m^2	1.34×10^{-3}	3.27×10^{-4}	7.93×10^{-5}	2.72×10^{-4}	-

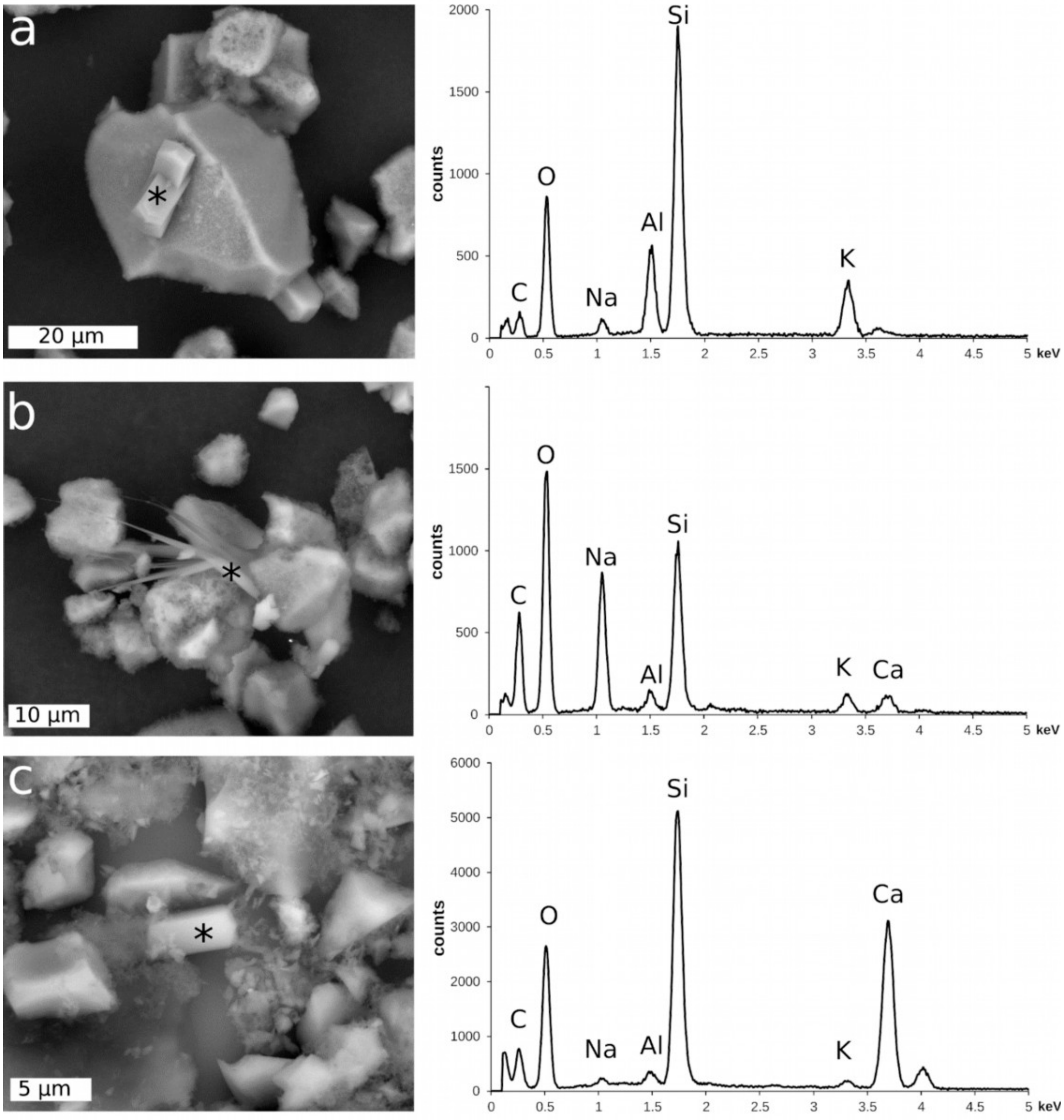

Figure S2. SEM image and corresponding EDX-spectrum of spot measurements marked with * of selected phases: (**a**) zeolite, (**b**) sodium aluminum silicate hydrate, and (**c**) calcite (note that the high Si peak in the EDS spectrum is due to the Si holder on which the sample was deposited).

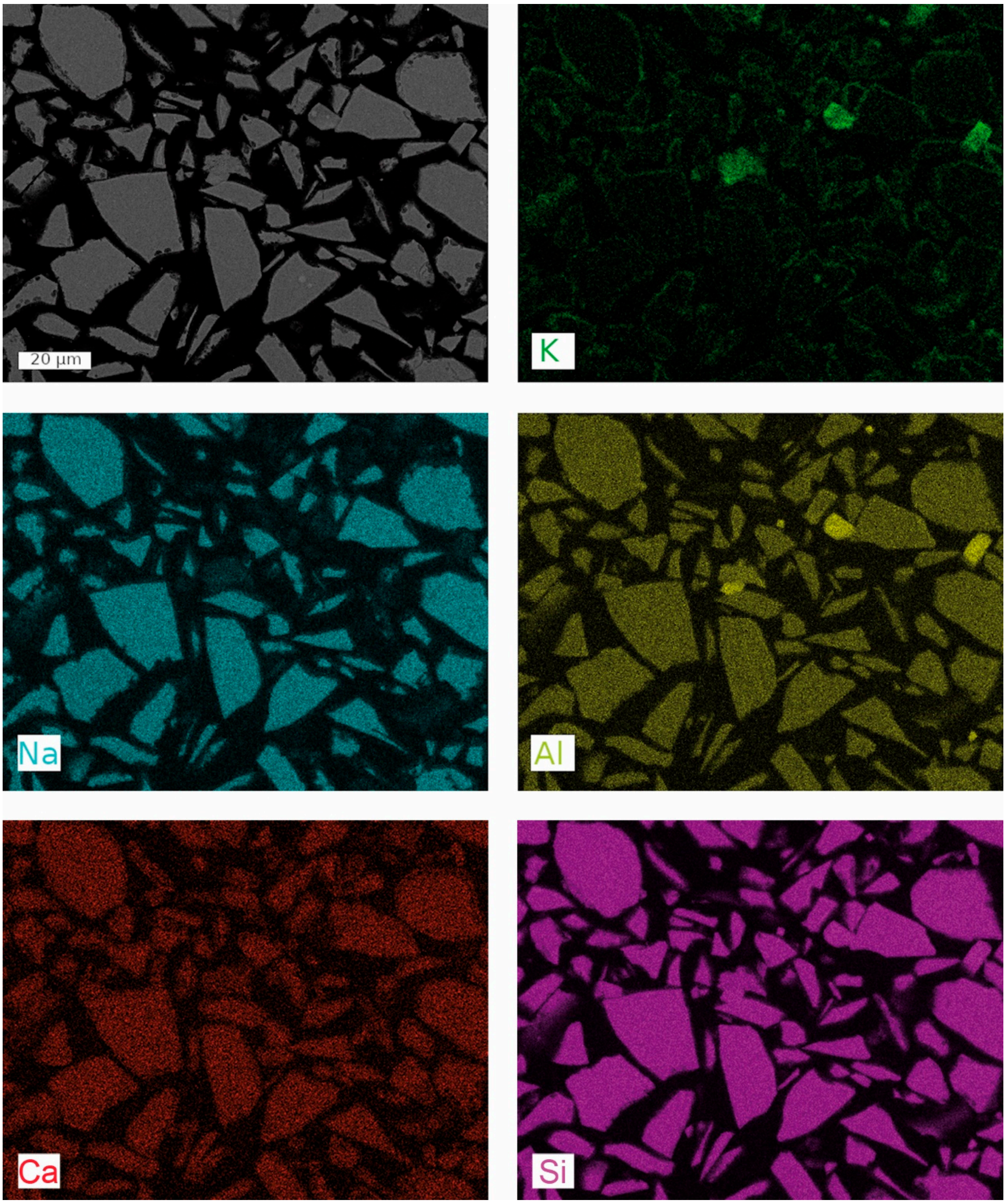

Figure S3. SEM-BSE image and corresponding elemental mappings of K-, Na-, Al-, Ca- and Si-K lines of embedded altered glass taken after 385 days in YCWCa at 70 °C.

Table 2. TEM-EDX spot measurements on altered glass; theoretical glass composition is provided for comparison.

Position TEM-EDX spot measurements	Na	Al	Si	K	Ca	Zr	B	Total	Al/Si Ratio
	wt.%								
bulk glass_1	3.5	3.5	30.5	-	3.0	2.3	-	100	0.12
bulk glass_2	7.0	3.4	29.0	-	3.5	1.6	-	100	0.12
altered (porous) layer	1.9	3.9	32.8	2.5	2.7	2.3	-	100	0.12
large pore	3.8	4.1	33.3	2.5	1.1	1.8	-	100	0.12
CSH_1	4.0	0.4	22.0	6.5	8.0	-	-	100	0.02
CSH_2	3.3	-	22.1	4.2	8.0	0.5	-	100	0.00
CSH_3	3.1	-	22.9	5.2	6.1	-	-	100	0.00
Theoretical glass composition	9.0	3.2	26.3	-	3.6	2.4	5.4	100	0.12
